# Isolation of two novel reassortant H3N6 avian influenza viruses from long‐distance migratory birds in Jiangxi Province, China

**DOI:** 10.1002/mbo3.1060

**Published:** 2020-05-28

**Authors:** Ruiyun Li, Tao Zhang, Jian Xu, Jianyu Chang, Bing Xu

**Affiliations:** ^1^ Department of Infectious Disease Epidemiology Faculty of Medicine MRC Centre for Global Infectious Disease Analysis School of Public Health Imperial College London London UK; ^2^ Ministry of Education Key Laboratory for Earth System Modelling Department of Earth System Science Tsinghua University Beijing China; ^3^ Centre for Healthy Cities Institute for China Sustainable Urbanization Tsinghua University Beijing China; ^4^ Ministry of Education's Key Laboratory of Poyang Lake Wetland and Watershed Research School of Geography and Environmental Science Jiangxi Normal University Nanchang China; ^5^ College of Veterinary Medicine China Agricultural University Beijing China

**Keywords:** avian influenza virus, China, H3N6, reassortant, wild birds

## Abstract

Two novel reassortant avian influenza A (H3N6) viruses were isolated from swan goose in Poyang Lake, Jiangxi Province, China, in 2014. Phylogenetic analyses indicated that these viruses are most likely derived from the Eurasian‐originated H3Ny (N3, N6, N8) and H5N6 viruses circulating among wild and domestic birds. It is noteworthy that H9N2 viruses have contributed PB1 gene to these novel H3N6 viruses. Our findings provide phylogenetic evidence to elucidate the ongoing viral reassortment in the wild bird population in southern China. Active surveillance of avian influenza viruses in Poyang Lake is warranted.

## Graphical abstract

Two avian influenza A/H3N6 viruses were isolated from a wild bird in Poyang Lake, Jiangxi, China, in 2014. These Jiangxi H3N6 viruses are novel reassortants with genes most likely derived from Eurasian‐originated H3Ny (N3, N6, N8), H5N6 viruses, and H9N2 viruses. We highlight the importance of routine surveillance in wild birds for a better early warning system of emerging reassortants with pandemic potential and consequently to avert the threat to public health.

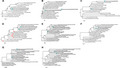

## 1 INTRODUCTION

The H3 viruses possess high adaptability to infect various hosts, ranging from birds to mammals (Bean et al., 1992). Among multiple cocirculating avian influenza virus (AIV) subtypes in domestic poultry in China, H3 viruses are one of the most frequently identified subtypes and have undergone extensive reassortments (Deng et al., 2013; Luo et al., 2017). Of note, some H3 viruses shared a close genetic relationship with highly pathogenetic H5N8 viruses (Cui et al., 2016). Additionally, H9N2 has undergone a constant change of genetics and pathogenicity since 1994 onward (Bi et al., 2011). By contributing their internal genes, H9N2 viruses have facilitated the emergence of novel reassortants such as H5N6, H7N9, and H10N8 (Bi et al., 2016; Liu, Li, et al., 2015; Liu, Xie, et al., 2015; Pu et al., 2015). This frequent reassortment raises the concern about its potential to have continuous reassortment with other subtypes such as H3. In the interim, constant surveillance in wild bird population has been carried out. Although substantial phylogenetic diversity has been proposed (Zhang, Li, Zhu, Chang, & Xu, 2019; author's unpublished results), the extent of reassortment and the potential emergence of novel strains are likely to be underestimated due primarily to the limited surveillance in wild birds.

## 2 MATERIALS AND METHODS

To elucidate a complete landscape of AIV ecology in wild birds, we undertook a routine bird ring survey in Poyang Lake, Jiangxi Province, in 2014–2016. Details of surveillance and isolation of AIVs have been presented in our previous studies (Zhang et al., 2019). Briefly, tracheal and cloacal swab samples were collected from migratory birds and domestic ducks and chickens. Virus isolation using these specimens was conducted in 9‐ to 11‐day‐old specific pathogen‐free embryonated chicken eggs. The viral RNAs were extracted from allantoic fluid of samples with hemagglutination activity using RNeasy Mini Kit (Qiagen), and reverse transcription was carried out using the SuperScript III Reverse Transcription‐PCR (RT‐PCR) Kit (Invitrogen). The subtype of these positive samples was determined using PCR of a marker gene (Tsukamoto et al., 2009; Lee, Chang, Shien, Cheng, & Shieh, 2001). All segments of these samples were amplified using a Phusion High‐Fidelity PCR System (New England Biolabs) (Hoffmann, Stech, Guan, Webster, & Perez, 2001) and sequenced as individual amplicons using Applied Biosystems Automated 3730xl DNA Analyzer.

Here, we report the isolation of two novel H3N6 reassortants from swan goose in 2014. They were designated as A/swan goose/Jiangxi/H23/2014(H3N6) (Gs/JX/H23) and A/swan goose/Jiangxi/H24/2014(H3N6) (Gs/JX/H24).

To investigate the phylogenetic relationship of Jiangxi H3N6 viruses with other AIV subtypes, we selected representative sequences by using the clustering algorithm and BLAST search. For each of the eight genes, we initially retrieved all the sequences available from GenBank and GISAID repositories. To reduce the size of this dataset, we firstly removed identical sequences by keeping the sequence with the earliest data. With the remaining sequences, we constructed the maximum‐likelihood tree and used the clustering algorithm to select representative sequences. Additionally, we used BLAST search to identify sequences with >90% identity. Sequences selected by clustering algorithm and BLAST search were integrated as a dataset of reference sequences (Tables A1, A2, A3, A4, A5, A6, A7, A8). Molecular phylogenetic analyses were conducted using the maximum‐likelihood method based on the Kimura 2‐parameter model (Kimura, 1980) with 1,000 bootstrap replicates in MEGA 6.0 software (Tamura, Stecher, Peterson, Filipski, & Kumar, 2013). Nucleotide substitution models were compared and selected based on BIC (Table A9). Phylogenetic relationship between H3N6 and other subtypes was investigated based on the topology of phylogenies, which was then verified by estimating the evolutionary divergence.

## 3 RESULTS AND DISCUSSION

A BLAST search in GenBank database showed that most of the gene segments of Jiangxi H3N6 viruses shared a high level of nucleotide identity with a local H5N6 strain, that is, A/chicken/Jiangxi/NCDZT1126/2014, with the identity over 93% (Table 1). Additionally, H3N6 viruses contain the closest HA gene to that of A/common shelduck/Mongolia/2076/2011(H3N8) with >98% identity. Of note, PB1 gene of Gs/JX/H23 and Gs/JX/H24 was closely related to an H9N2 strain, that is, A/chicken/Liaoning/1116/2012, with 98.46% and 98.63% identity, respectively.

**TABLE 1:** Nucleotide sequence identities between the Jiangxi H3N6 viruses and the closest homologs in the GenBank database

Gene	Virus	Accession	Subtype	Identity (%)	
				Gs/JX/H23	Gs/JX/H24
HA	A/common shelduck/Mongolia/2076/2011	KF501077	H3N8	98.77	98.82
NA	A/chicken/Jiangxi/NCDZT1126/2014	KP090449	H5N6	99.23	93.52
PB2	A/chicken/Jiangxi/NCDZT1126/2014	KP090444	H5N6	99.17	99.21
PB1	A/chicken/Liaoning/1116/2012	KM609826	H9N2	98.46	98.63
PA	A/chicken/Jiangxi/NCDZT1126/2014	KP090446	H5N6	99.87	99.51
NP	A/chicken/Jiangxi/NCDZT1126/2014	KP090445	H5N6	99.62	99.36
M	A/chicken/Jiangxi/NCDZT1126/2014	KP090450	H5N6	99.03	99.61
NS	A/chicken/Jiangxi/NCDZT1126/2014	KP090451	H5N6	99.51	98.79

**FIGURE 1:**
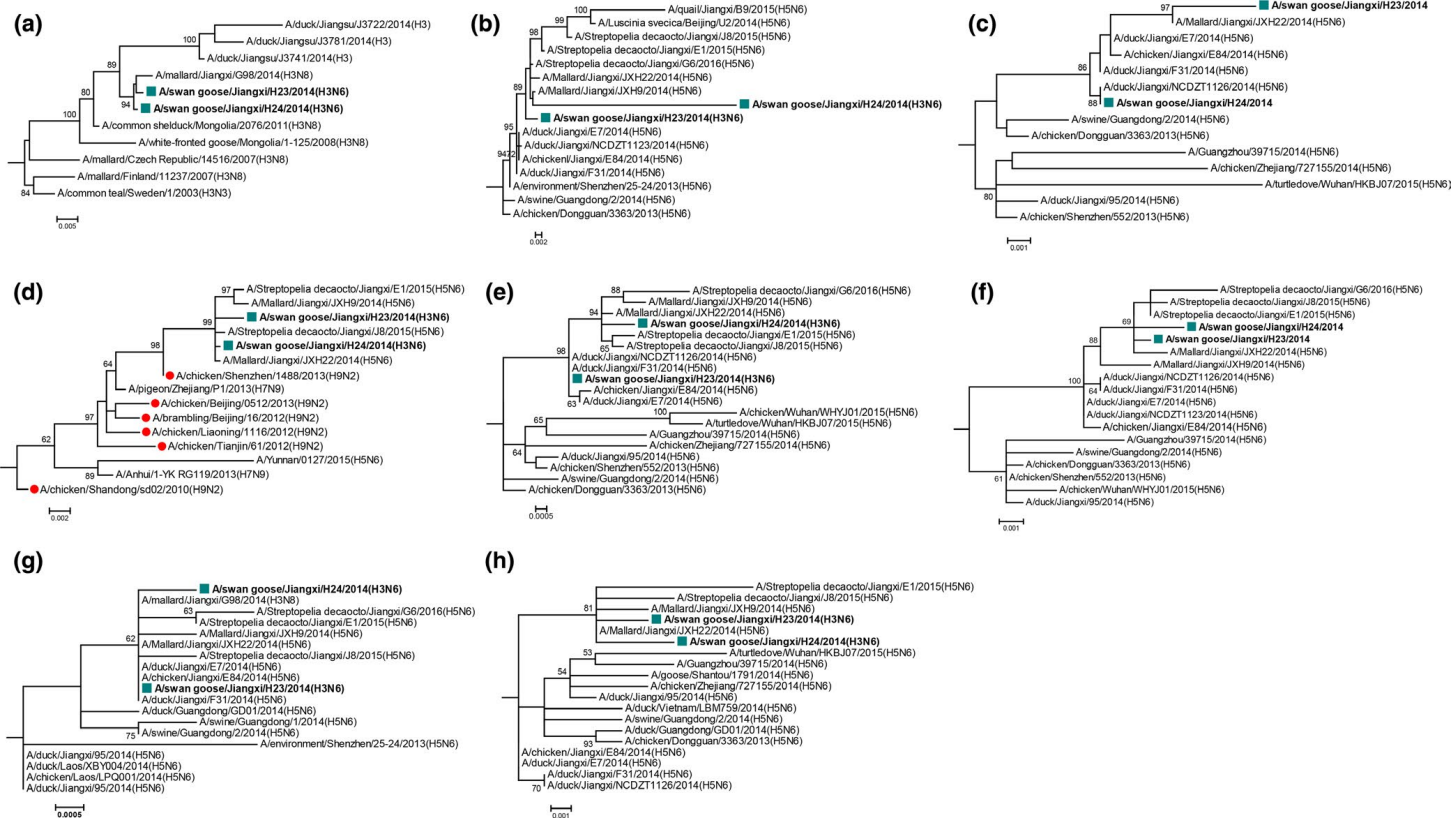
Phylogeny of all gene segments of Jiangxi H3N6 viruses. Phylogeny of the HA (A), NA (B), PB2 (C), PB1 (D), PA (E), NP (F), M(G), and NS (H) gene was constructed using the maximum‐likelihood method with 1,000 bootstrap replicates in MEGA v6.06. Jiangxi H3N6 viruses were highlighted by squares. H9N2 viruses were marked by red dots. Bootstrap values >70% were shown on the branches

**FIGURE A1:**
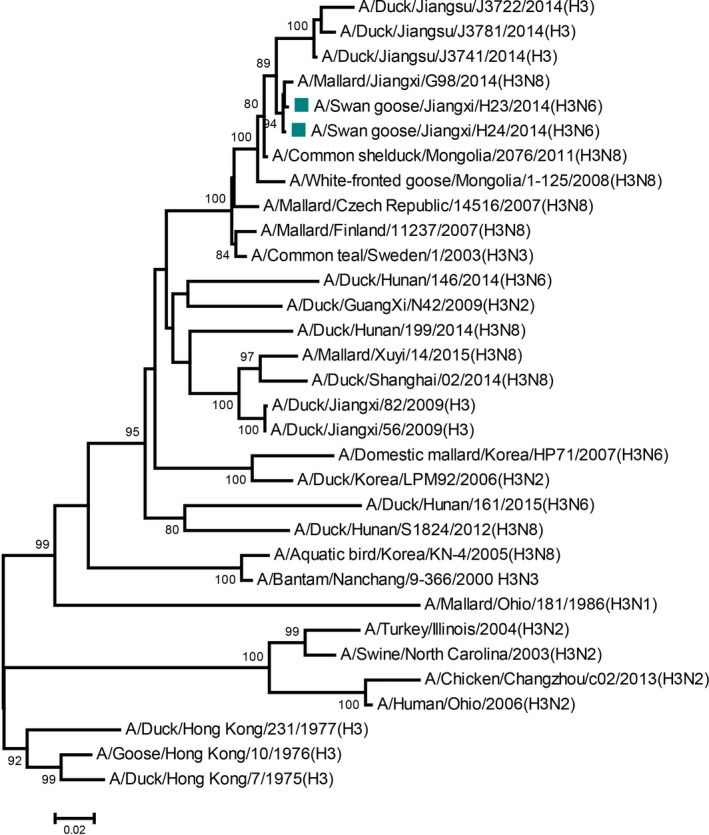
Phylogeny of HA gene of all analysed sequences. Phylogeny of the HA gene was constructed using the maximum likelihood method with 1,000 bootstrap replicates in MEGA v6.06. Jiangxi H3N6 viruses were highlighted by squares. Bootstrap values > 80% were shown on the branches

**FIGURE A2:**
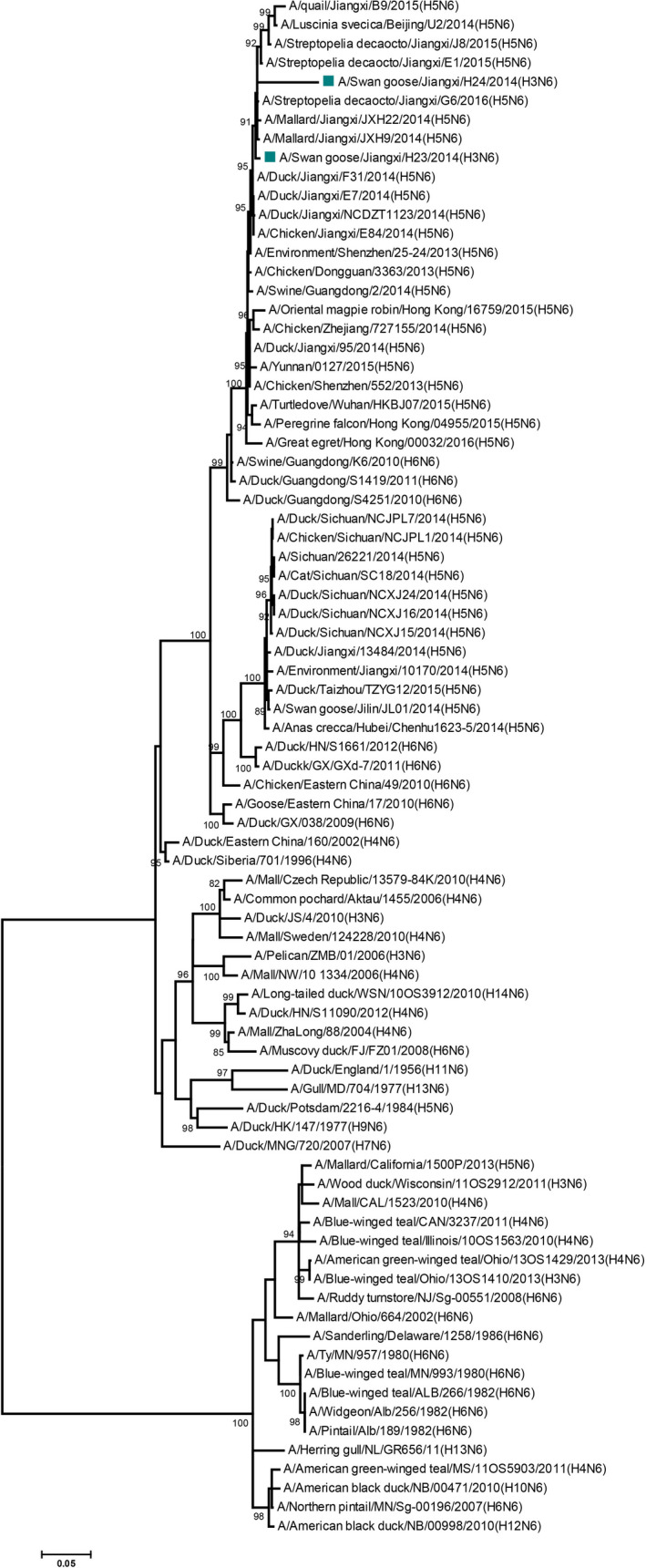
Phylogeny of NA gene of all analysed sequences. Phylogeny of the NA gene was constructed using the maximum likelihood method with 1,000 bootstrap replicates in MEGA v6.06. Jiangxi H3N6 viruses were highlighted by squares. Bootstrap values > 80% were shown on the branches

**FIGURE A3:**
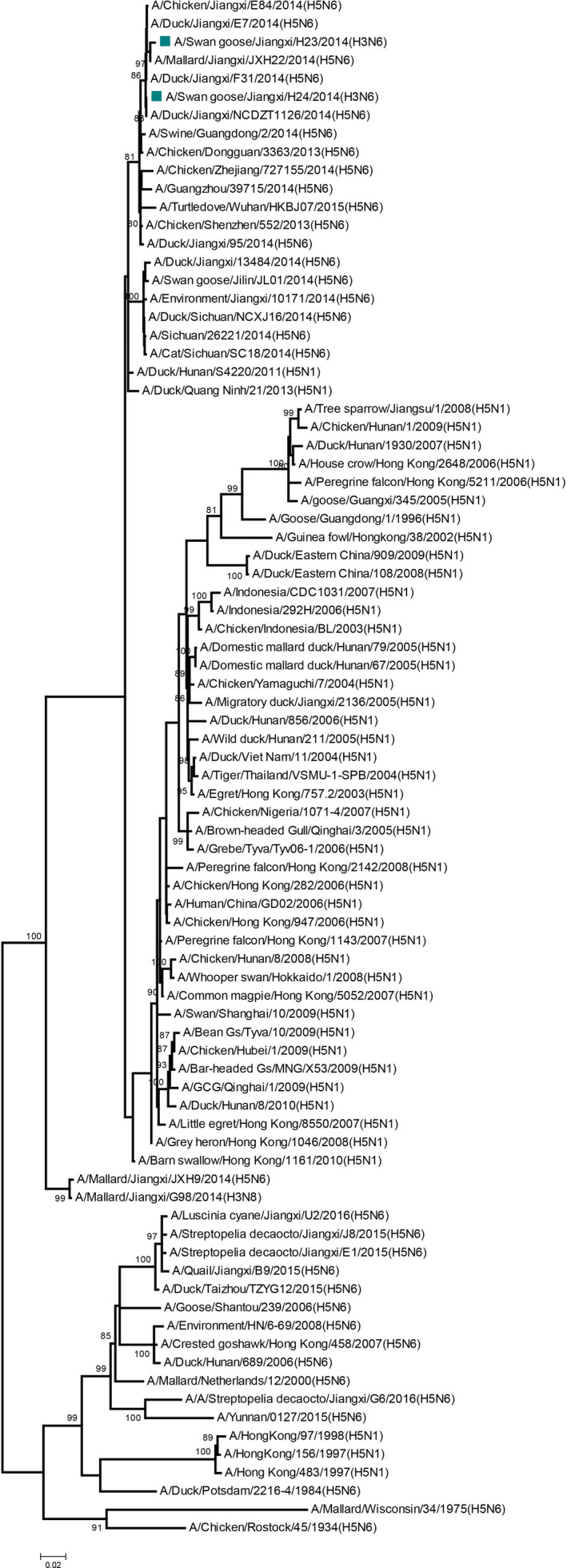
Phylogeny of PB2 gene of all analysed sequences. Phylogeny of the PB2 gene was constructed using the maximum likelihood method with 1,000 bootstrap replicates in MEGA v6.06. Jiangxi H3N6 viruses were highlighted by squares. Bootstrap values > 80% were shown on the branches

**FIGURE A4:**
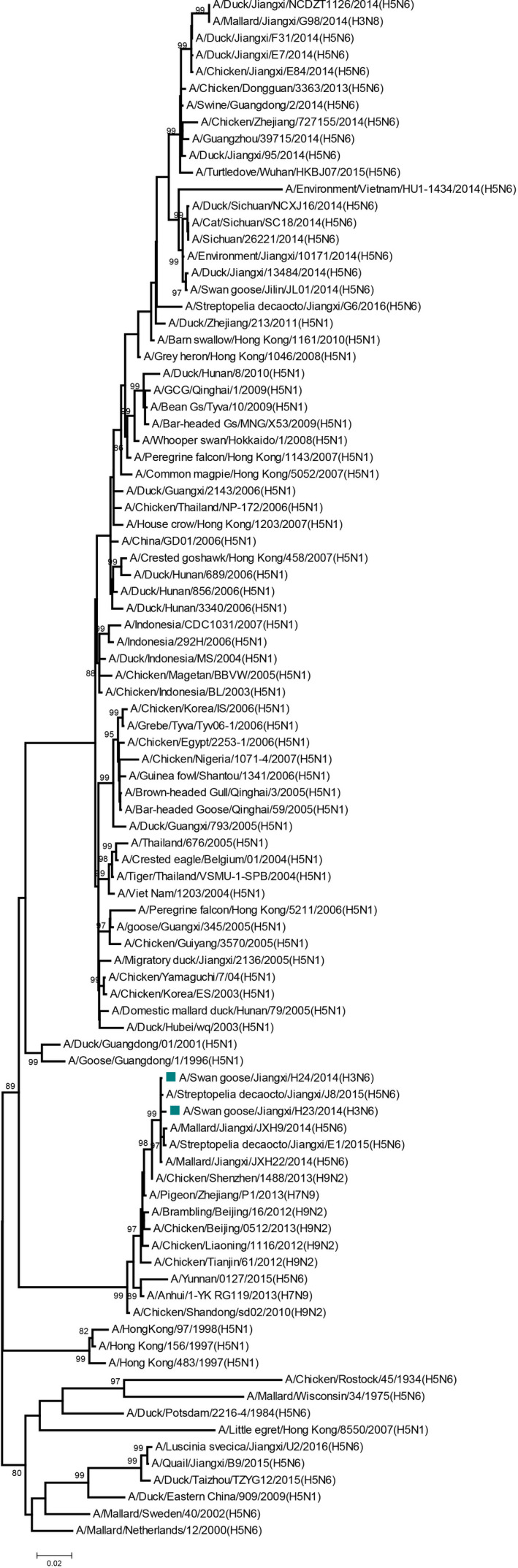
Phylogeny of PB1 gene of all analysed sequences. Phylogeny of the PB1 gene was constructed using the maximum likelihood method with 1,000 bootstrap replicates in MEGA v6.06. Jiangxi H3N6 viruses were highlighted by squares. Bootstrap values > 80% were shown on the branches

**FIGURE A5:**
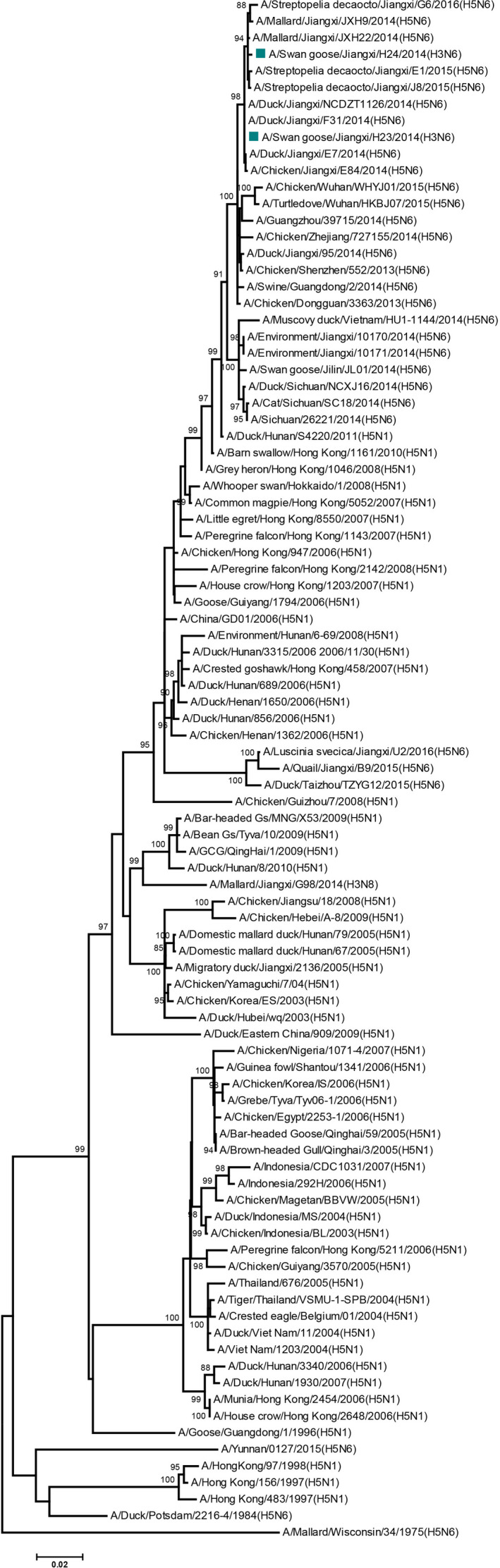
Phylogeny of the PA gene of all analysed sequences. Phylogeny of the PA gene was constructed using the maximum likelihood method with 1,000 bootstrap replicates in MEGA v6.06. Jiangxi H3N6 viruses were highlighted by squares. Bootstrap values > 80% were shown on the branches

**FIGURE A6:**
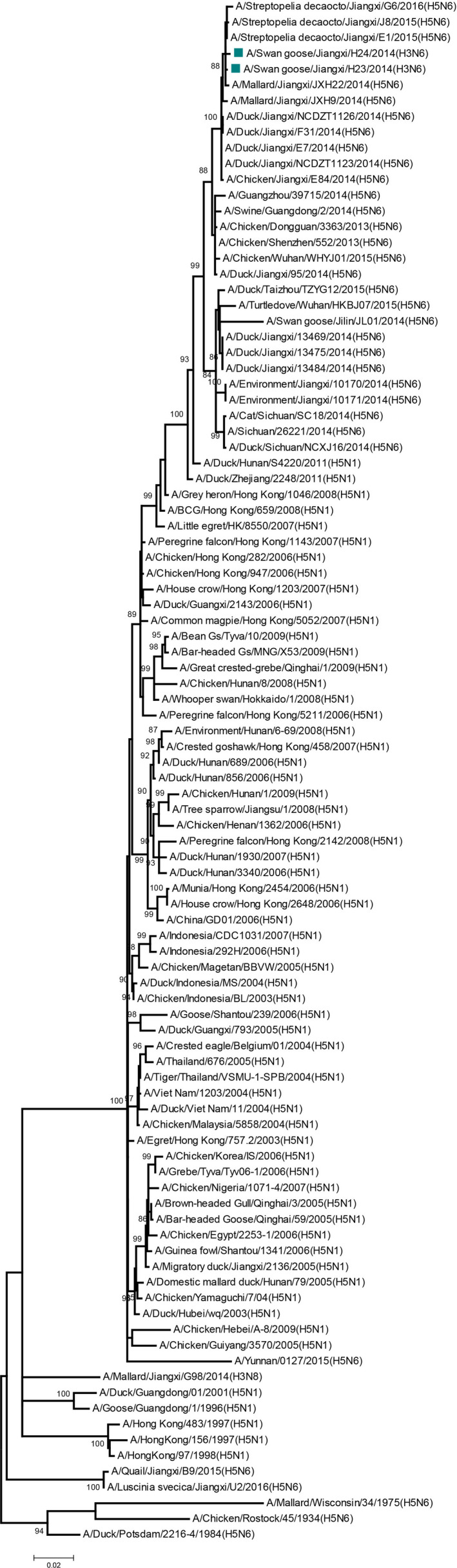
Phylogeny of NP gene of all analysed sequences. Phylogeny of the NP gene was constructed using the maximum likelihood method with 1,000 bootstrap replicates in MEGA v6.06. Jiangxi H3N6 viruses were highlighted by squares. Bootstrap values > 80% were shown on the branches

**FIGURE A7:**
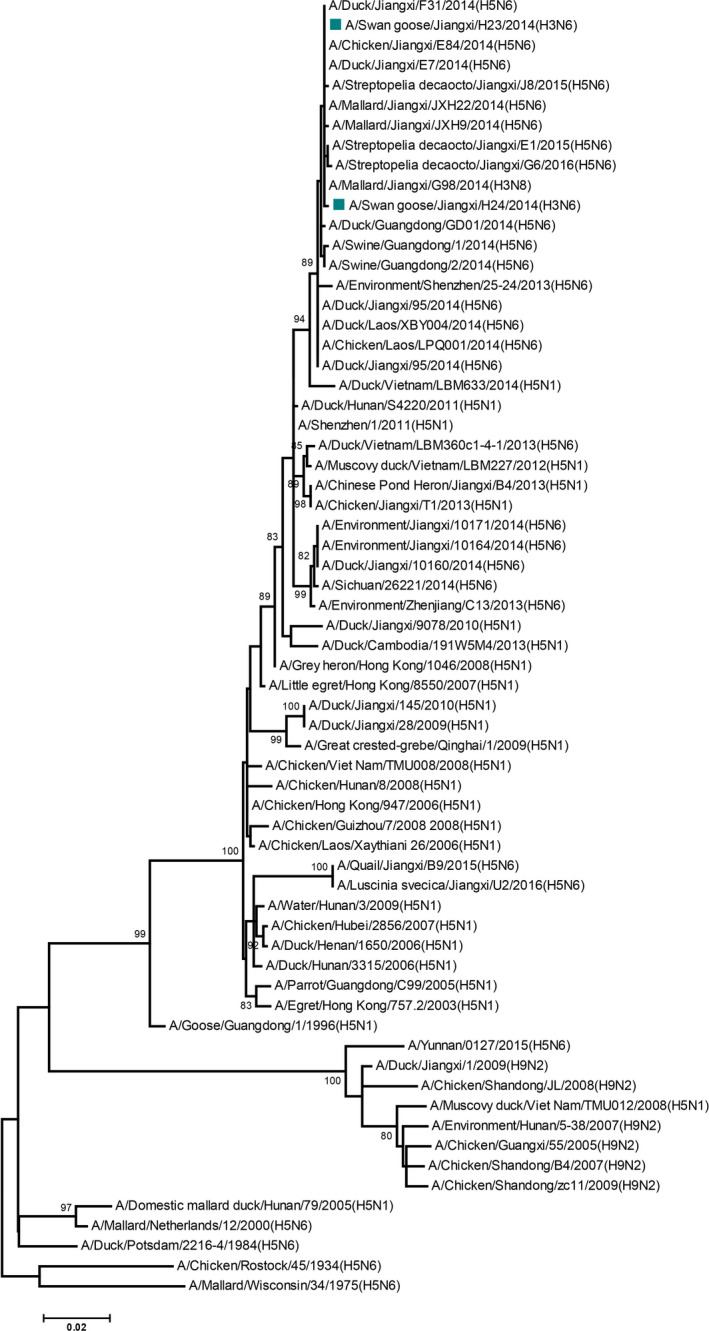
Phylogeny of M gene of all analysed sequences. Phylogeny of the M gene was constructed using the maximum likelihood method with 1,000 bootstrap replicates in MEGA v6.06. Jiangxi H3N6 viruses were highlighted by squares. Bootstrap values > 80% were shown on the branches

**FIGURE A8:**
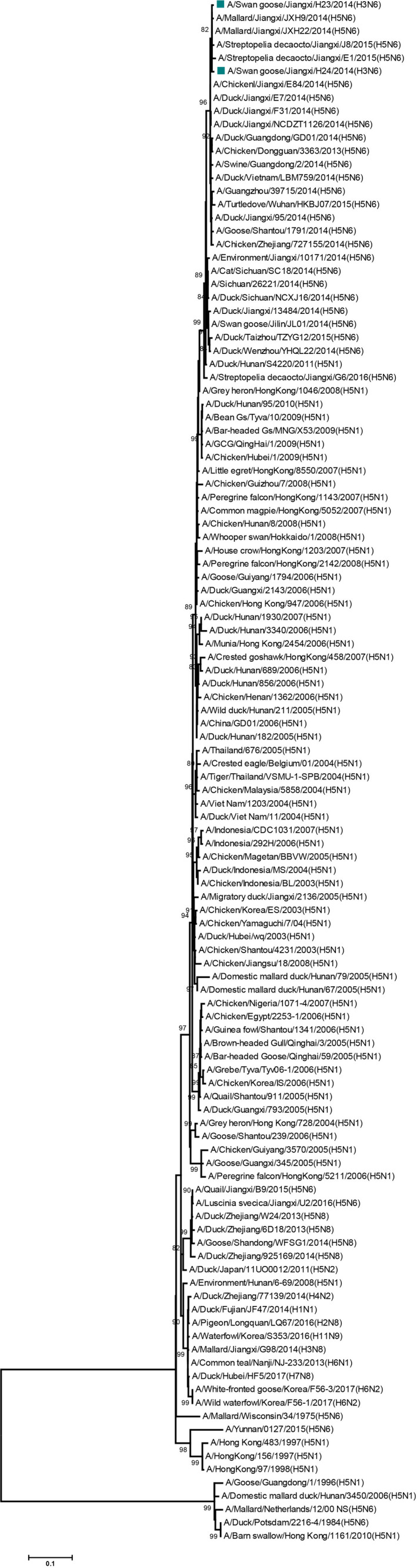
Phylogeny of NS gene of all analysed sequences. Phylogeny of the NS gene was constructed using the maximum likelihood method with 1,000 bootstrap replicates in MEGA v6.06. Jiangxi H3N6 viruses were highlighted by squares. Bootstrap values > 80% were shown on the branches

Phylogenetic analyses showed that all segments of Jiangxi H3N6 viruses belonged to the Eurasian lineage (Figures A1, A2, A3, A4, A5, A6, A7, A8). The phylogeny of surface genes revealed that Jiangxi H3N6 viruses are likely originated from H3Ny (N3, N6, N8) and H5N6 viruses. Most of their internal genes (except for PB1) are derived from recent H5N6 viruses isolated from both wild and domestic birds. Notably, PB1 gene is of the H9N2‐derived origin from domestic birds (Table 2). Also, the various origins possessed by novel Jiangxi H3N6 viruses identified in wild birds in Poyang Lake suggest the complex reassortment of AIV subtypes in China. It is acknowledged that cocirculation of different subtypes serves as the primary mechanism for the generation of novel variants through extensive gene reassortment. Among these variants, H5N6 and H9N2 were identified as two dominant AIV subtypes with dynamic reassortments among birds in China (Bi et al., 2016; Liu, Shi, & Gao, 2014). Our bird survey revealed the high prevalence of H5N6 viruses and mixed samples containing H5 and N6 genes (author's unpublished results), indicating the possibility that H5N6 may have greater compatibility with other subtypes within wild bird reservoir. As such, they are more likely to contribute their genes to generate novel strains such as the H3N6 viruses isolated in this study (Figure 1). Likewise, previous studies demonstrated that novel reassortant H3N6 viruses isolated from domestic ducks contain some gene segments from the dominant H5N6 viruses (Bi et al., 2016; Li et al., 2016; Liu, Li, et al., 2015; Liu, Xie, et al., 2015).

**TABLE 2:** Estimates of evolutionary divergence between Jiangxi H3N6 viruses and other prevailing viruses

Gene	Subtype	Pairwise genetic distance
NA	H5N6	0.049
PB2	H5N6	0.025
PB1	H5N6	0.009
	H9N2	0.016
PA	H5N6	0.01
NP	H5N6	0.013
M	H5N6	0.006
NS	H5N6	0.021

Note

The pairwise genetic distance was estimated by the average number of substitutions per site over all sequence pairs between H3N6 and H5N6/H9N2 viruses.

The genetic analysis strongly argues the crucial role that long‐distance migration birds have played in viral introduction and circulation at Poyang Lake, a migratory corridor situated within East Asian Flyway. Some wild bird species could carry AIVs over several hundred kilometers of their movement and shed viruses at stopover sites. Given the free‐range manner and limited biosafety measures of poultry raising in Poyang Lake, sharing common habitats such as the same waterbody and outdoor areas might have created opportunities for poultry to acquire viruses shed by migratory birds. Through frequent exposure and continuous reassortment, viruses isolated from wild and domestic birds share a high genetic relatedness which suggests a two‐way viral transmission at the wild–domestic interface.

## 4 CONCLUSIONS

In summary, two H3N6 reassortants were isolated from wild birds in Jiangxi Province, presumably originated from Eurasian H3Ny, H5N6, and H9N2 viruses. The findings suggested the complexity of reassortment of multiple AIV subtypes. Therefore, active surveillance in wild birds could improve the early warning system for an avian influenza outbreak.

## CONFLICT OF INTEREST

None declared.

## AUTHOR CONTRIBUTIONS

**Ruiyun Li:** Conceptualization (lead); formal analysis (lead); methodology (lead). **Tao Zhang:** Conceptualization (equal); formal analysis (equal); investigation (equal). **Jian Xu:** Investigation (equal). **Jianyu Chang:** Investigation (equal). **Bing Xu:** Funding acquisition (lead).

## ETHICAL STATEMENT

All animal work was approved by the Beijing Association for Science and Technology (approval SYXK [Beijing] 2007‐0023). The laboratory animal research was performed in the microbiology laboratory of China Agricultural University, following the Beijing Laboratory Animal Welfare and Ethics guidelines of the Beijing Administration Committee of Laboratory Animals and China Agricultural University Institutional Animal Care and Use Committee guidelines (ID: SKLAB‐B‐2010‐003).

## Data Availability

Sequences were deposited in GenBank under the accession numbers MT375330–MT375337 and MT375532–MT375539.

## APPENDIX A

**TABLE A1:** Details of sequences of HA gene analysed in this study

Viruses name	Accession	Viruses name	Accession
A/domestic mallard/Korea/HP71/2007(H3N6)	JN087216	A/Bantam/Nanchang/9‐366/2000(H3N3)	CY006012
A/human/Ohio/2006(H3N2)	EF554795	A/mallard/Ohio/181/1986(H3N1)	CY021429
A/DK/HK/7/1975(H3)	D00929	A/duck/Jiangxi/56/2009(H3)	unpublished
A/duck/Hong Kong/231/77(H3)	D00932	A/duck/Jiangxi/82/2009(H3)	unpublished
A/Gs/HK/10/1976(H3)	D00930	A/swan goose/Jiangxi/H24/2014(H3N6)	MT375535
A/swine/North Carolina/00116/2003(H3N2)	CY195532	A/swan goose/Jiangxi/H23/2014(H3N6)	MT375333
A/turkey/Illinois/2004(H3N2)	EF551045	A/mallard/Jiangxi/G98/2014(H3N8)	MN473213
A/duck/Korea/LPM92/2006(H3N2)	EU301210	A/chicken/Changzhou/c02/2013(H3N2)	EPI556441*
A/aquatic bird/Korea/KN‐4/2005(H3N8)	EU301219	A/duck/Shanghai/02/2014(H3N8)	KR703241
A/duck/GuangXi/N42/2009(H3N2)	JN003630	A/duck/Jiangsu/J3722/2014(H3)	KP767634
A/common shelduck/Mongolia/2076/2011(H3N8)	KF501077	A/duck/Jiangsu/J3741/2014(H3)	KP767635
A/white‐fronted goose/Mongolia/1‐125/2008(H3N8)	JN029680	A/duck/Jiangsu/J3781/2014(H3)	KP767636
A/common teal/Sweden/1/2003(H3N3)	CY060185	A/duck/Hunan/199/2014(H3N8)	KX121246
A/mallard/Finland/11237/2007(H3N8)	KF183636	A/duck/Hunan/146/2014(H3N6)	KX121254
A/mallard/Czech_Republic/14516/2007(H3N8)	JF682614	A/duck/Hunan/161/2015(H3N6)	KX121262
A/duck/Hunan/S1824/2012(H3N8)	CY146628	A/mallard/Xuyi/14/2015(H3N8)	KX160095

* Data source: GISAID.

**TABLE A2:** Details of sequences of NA gene analysed in this study

Viruses name	Accession	Viruses name	Accession
A/environment/Shenzhen/25‐24/2013(H5N6)	EPI530065*	A/long‐tailed duck/Wisconsin/10OS3912/2010(H14N6)	KC110598
A/Muscovy duck/Fujian/FZ01/2008(H6N6)	GU595163	A/herring gull/Newfoundland/GR656/2011(H13N6)	KC845092
A/northern pintail/Minnesota/Sg‐00196/2007(H6N6)	CY042177	A/duck/Potsdam/2216‐4/1984(H5N6)	CY005771
A/blue‐winged teal/ALB/266/1982(H6N6)	EPI85891*	A/swan goose/Jilin/JL01/2014(H5N6)	KM873648
A/blue‐winged teal/MN/993/80(H6N6)	CY005883	A/duck/Jiangxi/NCDZT1123/2014(H5N6)	KP090442
A/duck/Guangxi/038/2009(H6N6)	HQ599859	A/chicken/Shenzhen/552/2013(H5N6)	KP284967
A/mallard/Ohio/664/2002(H6N6)	CY020855	A/environment/Jiangxi/10170/2014(H5N6)	KP286463
A/pintail/Alb/189/1982(H6N6)	CY004148	A/duck/Jiangxi/13484/2014(H5N6)	KP286439
A/ruddy turnstone/New Jersey/Sg‐00551/2008(H6N6)	CY145665	A/chicken/Dongguan/3363/2013(H5N6)	KP286111
A/sanderling/Delaware/1258/86(H6N6)	AY207557	A/swine/Guangdong/2/2014(H5N6)	KT313412
A/turkey/Minnesota/957/80(H6N6)	AY207547	A/chicken/Zhejiang/727155/2014(H5N6)	KU042824
A/widgeon/Alb/256/1982(H6N6)	CY004156	A/turtledove/Wuhan/HKBJ07/2015(H5N6)	KU143358
A/duck/Eastern China/160/2002(H4N6)	EU429706	A/duck/Taizhou/TZYG12/2015(H5N6)	KU143369
A/mallard/ZhaLong/88/2004(H4N6)	FJ349248	A/Yunnan/0127/2015(H5N6)	KT245145
A/mallard/Norway/10_1334/2006(H4N6)	FM179763	A/cat/Sichuan/SC18/2014(H5N6)	KM873640
A/pelican/Zambia/01/2006(H3N6)	AB470298	A/duck/Guangdong/S1419/2011(H6N6)	KJ200743
A/Common_pochard/Aktau/1455/2006(H4N6)	FJ434372	A/duck/Guangdong/S4251/2010(H6N6)	KJ200823
A/duck/Mongolia/720/2007(H7N6)	AB450449	A/Anas_crecca/Hubei/Chenhu1623‐5/2014(H5N6)	KM251485
A/duck/HK/147/1977(H9N6)	CY005641	A/chicken/Sichuan/NCJPL1/2014(H5N6)	KM251486
A/gull/Maryland/704/1977(H13N6)	CY130088	A/duck/Sichuan/NCJPL7/2014(H5N6)	KM251487
A/duck/England/1/1956(H11N6)	GQ247863	A/duck/Sichuan/NCXJ15/2014(H5N6)	KM251488
A/duck/Siberia/701/1996(H4N6)	AB292663	A/duck/Sichuan/NCXJ16/2014(H5N6)	KM251489
A/swine/Guangdong/K6/2010(H6N6)	HM804474	A/duck/Sichuan/NCXJ24/2014(H5N6)	KM251490
A/chicken/Eastern China/49/2010(H6N6)	JF965320	A/peregrine_falcon/Hong_Kong/04955/2015(H5N6)	EPI687145*
A/duck/Guangxi/GXd‐7/2011(H6N6)	JX304772	A/great_egret/Hong_Kong/00032/2016(H5N6)	EPI687157*
A/duck/Hunan/S11090/2012(H4N6)	CY146550	A/Sichuan/26221/2014(H5N6)	EPI533584*
A/duck/Hunan/S1661/2012(H6N6)	CY146622	A/mallard/California/1500P/2013(H5N6)	CY177427
A/duck/Jiangsu/4/2010(H3N6)	KC261679	A/quail/Jiangxi/B9/2015(H5N6)	EPI1574331*
A/goose/Eastern China/17/2010(H6N6)	JF965334	A/Streptopelia decaocto/Jiangxi/E1/2015(H5N6)	EPI1574315*
A/blue‐winged teal/Ohio/13OS1410/2013(H3N6)	KJ526082	A/Streptopelia decaocto/Jiangxi/G6/2016(H5N6)	EPI1489550*
A/blue‐winged teal/Illinois/10OS1563/2010(H4N6)	CY132911	A/Mallard/Jiangxi/JXH9/2014(H5N6)	EPI1574347*
A/blue‐winged teal/Canada/3237/2011(H4N6)	CY149670	A/Mallard/Jiangxi/JXH22/2014(H5N6)	EPI1574339*
A/American Green‐winged Teal/Ohio/18OS01750/2018(H4N6)	MN431022	A/Streptopelia decaocto/Jiangxi/J8/2015(H5N6)	EPI1574323*
A/American green‐winged teal/Mississippi/11OS5903/2011(H4N6)	CY166834	A/Luscinia cyane/Jiangxi/U2/2016(H5N6)	EPI1574307*
A/American black duck/New Brunswick/00998/2010(H12N6)	CY139552	A/duck/Jiangxi/95/2014(H5N6)	EPI530056*
A/American black duck/New Brunswick/00471/2010(H10N6)	CY138908	A/duck/Jiangxi/E7/2014(H5N6)	Unpublished
A/wood duck/Wisconsin/11OS2912/2011(H3N6)	CY167112	A/chicken/Jiangxi/E84/2014(H5N6)	Unpublished
A/mallard/Sweden/124228/2010(H4N6)	CY166082	A/duck/Jiangxi/F31/2014(H5N6)	Unpublished
A/mallard/Czech Republic/13579‐84K/2010(H4N6)	JF789620	A/swan goose/Jiangxi/H24/2014(H3N6)	MT375537
A/mallard/California/1523/2010(H4N6)	CY120581	A/swan goose/Jiangxi/H23/2014(H3N6)	MT375335

* Data source: GISAID.

**TABLE A3:** Details of sequences of PB2 gene analysed in this study

Viruses name	Accession	Viruses name	Accession
A/Brown‐headed Gull/Qinghai/3/05(H5N1)	DQ095756	A/peregrine falcon/Hong Kong/5211/2006(H5N1)	CY036042
A/chicken/Hong Kong/282/2006(H5N1)	DQ992572	A/chicken/Hunan/8/2008(H5N1)	GU182155
A/chicken/Nigeria/1071‐4/2007(H5N1)	EU148379	A/duck/Eastern China/108/2008(H5N1)	GU727666
A/chicken/Yamaguchi/7/2004(H5N1)	AB166859	A/duck/Eastern China/909/2009(H5N1)	GU727674
A/Dk/Viet Nam/11/2004(H5N1)	AY651729	A/swan/Shanghai/10/2009(H5N1)	JF975558
A/domestic green‐winged teal/Hunan/67/2005(H5N1)	EU430499	A/tree sparrow/Jiangsu/1/2008(H5N1)	GQ202214
A/domestic mallard duck/Hunan/79/2005(H5N1)	EU430505	A/chicken/Rostock/45/1934(H7N1)	CY077417
A/duck/Hunan/856/2006(H5N1)	DQ992627	A/duck/Hunan/S4220/2011(H5N1)	CY146697
A/egret/Hong Kong/757.2/2003(H5N1)	AY676022	A/barn swallow/Hong Kong/1161/2010(H5N1)	KF735641
A/Gf/HK/38/2002(H5N1)	AY651457	A/duck/Potsdam/2216‐4/1984(H5N6)	CY005776
Goose/Guangdong/1/1996(H5N1)	NC007362	A/mallard/Wisconsin/34/1975(H5N6)	GU052005
A/grebe/Tyva/Tyv06‐1/2006(H5N1)	DQ914807	A/cat/Sichuan/SC18/2014(H5N6)	KM873635
A/HongKong/156/97(H5N1)	AF036363	A/swan goose/Jilin/JL01/2014(H5N6)	KM873643
A/Hong Kong/483/1997(H5N1)	GU052097	A/duck/Jiangxi/NCDZT1126/2014(H5N6)	KP090444
A/Hong Kong/97/98(H5N1)	AF258846	A/duck/Sichuan/NCXJ16/2014(H5N6)	KM251536
A/house crow/Hong Kong/2648/2006(H5N1)	DQ992568	A/chicken/Shenzhen/552/2013(H5N6)	KP284962
A/human/China/GD02/2006(H5N1)	EU263988	A/environment/Jiangxi/10171/2014(H5N6)	KP285050
A/Indonesia/292H/2006(H5N1)	EU146720	A/duck/Jiangxi/13484/2014(H5N6)	KP286434
A/Indonesia/CDC1031/2007(H5N1)	CY019349	A/chicken/Dongguan/3363/2013(H5N6)	KP286106
A/Tiger/Thailand/VSMU‐1‐SPB/2004(H5N1)	EF178522	A/swine/Guangdong/2/2014(H5N6)	KT313417
A/duck/Hunan/689/2006(H5N1)	FJ784825	A/chicken/Zhejiang/727155/2014(H5N6)	KU042679
A/Duck/Hunan/1930/2007(H5N1)	FJ784832	A/turtledove/Wuhan/HKBJ07/2015(H5N6)	KU143569
A/Mallard/Netherlands/12/2000(H7N3)	KF695236	A/duck/Taizhou/TZYG12/2015(H5N6)	KU143581
A/chicken/Hong Kong/947/2006 (H5N1)	DQ992577	A/Guangzhou/39715/2014(H5N6)	KP765785
A/goose/Guangxi/345/2005(H5N1)	DQ320830	A/Yunnan/0127/2015(H5N6)	KT245150
A/goose/Shantou/239/2006(H5N1)	DQ992683	A/Sichuan/26221/2014(H5N6)	EPI533585
A/migratory duck/Jiangxi/2136/2005(H5N1)	DQ320853	A/quail/Jiangxi/B9/2015(H5N6)	EPI1574326*
A/wild duck/Hunan/211/2005(H5N1)	EU329189	A/Streptopelia decaocto/Jiangxi/E1/2015(H5N6)	EPI1574310*
A/duck/Hunan/8/2010(H5N1)	GU182163	A/Mallard/Jiangxi/JXH9/2014(H5N6)	EPI1574342*
A/chicken/Hunan/1/2009(H5N1)	HM172425	A/Mallard/Jiangxi/JXH22/2014(H5N6)	EPI1574334*
A/great crested‐grebe/Qinghai/1/2009(H5N1)	CY063315	A/Streptopelia decaocto/Jiangxi/J8/2015(H5N6)	EPI1574318*
A/bar‐headed goose/Mongolia/X53/2009(H5N1)	KR732547	A/Luscinia cyane/Jiangxi/U2/2016(H5N6)	EPI1574302*
A/bean goose/Tyva/10/2009(H5N1)	GQ386154	A/Streptopelia decaocto/Jiangxi/G6/2016(H5N6)	EPI1489545*
A/environment/Hunan/6‐69/2008(H5N1)	GU182179	A/duck/Jiangxi/95/2014(H5N6)	EPI530051*
A/grey heron/Hong Kong/1046/2008(H5N1)	CY036242	A/duck/Jiangxi/E7/2014(H5N6)	Unpublished
A/whooper swan/Hokkaido/1/2008(H5N1)	AB436547	A/chicken/Jiangxi/E84/2014(H5N6)	Unpublished
A/common magpie/Hong Kong/5052/2007(H5N1)	CY036170	A/duck/Jiangxi/F31/2014(H5N6)	Unpublished
A/crested goshawk/Hong Kong/458/2007(H5N1)	CY036058	A/mallard/Jiangxi/G98/2014(H3N8)	MN473210
A/little egret/Hong Kong/8550/2007(H5N1)	CY036194	A/swan goose/Jiangxi/H24/2014(H3N6)	MT375532
A/peregrine falcon/Hong Kong/1143/2007(H5N1)	CY036090	A/swan goose/Jiangxi/H23/2014(H3N6)	MT375330
A/peregrine falcon/Hong Kong/2142/2008(H5N1)	CY036266		

* Data source: GISAID.

**TABLE A4:** Details of sequences of PB1 gene analysed in this study

Viruses name	Accession	Viruses name	Accession
A/Viet Nam/1203/2004(H5N1)	HM006757	A/peregrine falcon/Hong Kong/1143/2007(H5N1)	CY036091
A/Bar‐headed Goose/Qinghai/59/05(H5N1)	DQ095732	A/peregrine falcon/Hong Kong/5211/2006(H5N1)	CY036043
A/Brown‐headed Gull/Qinghai/3/05(H5N1)	DQ095736	A/duck/Eastern China/909/2009(H5N1)	GU727675
A/chicken/Egypt/2253‐1/2006(H5N1)	CY020651	A/barn swallow/Hong Kong/1161/2010(H5N1)	KF735642
A/chicken/Guiyang/3570/2005(H5N1)	EF124006	A/duck/Zhejiang/213/2011(H5N1)	JN646699
A/chicken/Korea/IS/2006(H5N1)	EU233681	A/duck/Potsdam/2216‐4/1984(H5N6)	CY005775
A/chicken/Magetan/BBVW/2005(H5N1)	DQ493343	A/mallard/Wisconsin/34/1975(H5N6)	GU052004
A/chicken/Nigeria/1071‐4/2007(H5N1)	EU148378	A/mallard/Sweden/40/2002(H5N6)	KF695348
A/chicken/Thailand/NP‐172/2006(H5N1)	DQ999875	A/cat/Sichuan/SC18/2014(H5N6)	KM873636
A/chicken/Yamaguchi/7/2004(H5N1)	GU186714	A/swan goose/Jilin/JL01/2014(H5N6)	KM873644
A/China/GD01/2006(H5N1)	DQ835311	A/duck/Jiangxi/NCDZT1126/2014(H5N6)	KP090445
A/crested eagle/Belgium/01/2004(H5N1)	DQ454102	A/duck/Sichuan/NCXJ16/2014(H5N6)	KM251526
A/duck/Indonesia/MS/2004(H5N1)	AY651652	A/environment/Jiangxi/10171/2014(H5N6)	KP285051
A/domestic mallard duck/Hunan/79/2005(H5N1)	EU430509	A/duck/Jiangxi/13484/2014(H5N6)	KP286435
A/duck/Guangdong/01/2001(H5N1)	AY585487	A/chicken/Dongguan/3363/2013(H5N6)	KP286107
A/duck/Guangxi/793/2005(H5N1)	DQ321294	A/swine/Guangdong/2/2014(H5N6)	KT313416
A/duck/Guangxi/2143/2006(H5N1)	EF124037	A/environment/Vietnam/HU1‐1434/2014(H5N6)	LC069866
A/duck/Hubei/wq/2003(H5N1)	DQ997177	A/chicken/Zhejiang/727155/2014(H5N6)	KU042708
A/duck/Hunan/856/2006(H5N1)	EF123953	A/turtledove/Wuhan/HKBJ07/2015(H5N6)	KU143527
A/goose/Guangdong/1/1996(H5N1)	NC007358	A/duck/Taizhou/TZYG12/2015(H5N6)	KU143538
A/grebe/Tyva/Tyv06‐1/2006(H5N1)	DQ914810	A/Guangzhou/39715/2014(H5N6)	KP765786
A/environment/Hong Kong/156/1997(H5N1)	GU052133	A/Yunnan/0127/2015(H5N6)	KT245149
A/Hong Kong/483/1997(H5N1)	GU052103	A/Sichuan/26221/2014(H5N6)	EPI533586*
A/Hong Kong/97/98(H5N1)	AF258827	A/chicken/Shenzhen/1488/2013(H9N2)	KP414379
A/Indonesia/292H/2006(H5N1)	EU146719	A/chicken/Liaoning/1116/2012(H9N2)	KM609826
A/Indonesia/CDC1031/2007(H5N1)	CY019350	A/chicken/Beijing/0512/2013(H9N2)	KM609836
A/Thailand/676/2005(H5N1)	DQ360838	A/pigeon/Zhejiang/P1/2013(H7N9)	KF042093
A/Tiger/Thailand/VSMU‐1‐SPB/2004(H5N1)	EF178523	A/chicken/Tianjin/61/2012(H9N2)	KF059336
A/duck/Hunan/689/2006(H5N1)	FJ784809	A/brambling/Beijing/16/2012(H9N2)	KC464596
A/duck/Hunan/3340/2006(H5N1)	FJ784824	A/chicken/Shandong/sd02/2010(H9N2)	KC821082
Mallard/Netherlands/12/2000(H7N3)	KF695237	A/Anhui/1‐YK RG119/2013(H7N9)	CY192601
A/goose/Guangxi/345/2005(H5N1)	DQ321291	A/quail/Jiangxi/B9/2015(H5N6)	EPI1574327*
A/Guinea fowl/Shantou/1341/2006(H5N1)	EF124007	A/Streptopelia decaocto/Jiangxi/E1/2015(H5N6)	EPI1574311*
A/migratory duck/Jiangxi/2136/2005(H5N1)	DQ321314	A/Streptopelia decaocto/Jiangxi/G6/2016(H5N6)	EPI1489546*
A/chicken/Rostock/45/1934(H7N1)	CY077418	A/Mallard/Jiangxi/JXH9/2014(H5N6)	EPI1574343*
A/duck/Hunan/8/2010(H5N1)	GU182164	A/Mallard/Jiangxi/JXH22/2014(H5N6)	EPI1574335*
A/great crested‐grebe/Qinghai/1/2009(H5N1)	CY063316	A/Streptopelia decaocto/Jiangxi/J8/2015(H5N6)	EPI1574319*
A/bar‐headed goose/Mongolia/X53/2009(H5N1)	KR732560	A/Luscinia cyane/Jiangxi/U2/2016(H5N6)	EPI1574303*
A/bean goose/Tyva/10/2009(H5N1)	GQ386155	A/duck/Jiangxi/95/2014(H5N6)	EPI530052*
A/grey heron/Hong Kong/1046/2008(H5N1)	CY036243	A/duck/Jiangxi/E7/2014(H5N6)	Unpublished
A/whooper swan/Hokkaido/1/2008(H5N1)	AB436548	A/chicken/Jiangxi/E84/2014(H5N6)	Unpublished
A/common magpie/Hong Kong/5052/2007(H5N1)	CY036171	A/duck/Jiangxi/F31/2014(H5N6)	Unpublished
A/crested goshawk/Hong Kong/458/2007(H5N1)	CY036059	A/swan goose/Jiangxi/H24/2014(H3N6)	MT375533
A/house crow/Hong Kong/1203/2007(H5N1)	CY036099	A/swan goose/Jiangxi/H23/2014(H3N6)	MT375331
A/little egret/Hong Kong/8550/2007(H5N1)	CY036195	A/mallard/Jiangxi/G98/2014(H3N8)	MN473211

* Data source: GISAID.

**TABLE A5:** Details of sequences of PA gene analysed in this study

Viruses name	Accession	Viruses name	Accession
A/Viet Nam/1203/2004(H5N1)	HM006758	A/crested goshawk/Hong Kong/458/2007(H5N1)	CY036060
A/Bar‐headed Goose/Qinghai/59/05(H5N1)	DQ095712	A/house crow/Hong Kong/1203/2007(H5N1)	CY036100
A/Brown‐headed Gull/Qinghai/3/05(H5N1)	DQ095716	A/peregrine falcon/Hong Kong/2142/2008(H5N1)	CY036268
A/chicken/Egypt/2253‐1/2006(H5N1)	CY020650	A/peregrine falcon/Hong Kong/5211/2006(H5N1)	CY036044
A/chicken/Guiyang/3570/2005(H5N1)	EF124761	A/little egret/Hong Kong/8550/2007(H5N1)	CY036196
A/chicken/Korea/IS/2006(H5N1)	EU233680	A/peregrine falcon/Hong Kong/1143/2007(H5N1)	CY036092
A/chicken/Magetan/BBVW/2005(H5N1)	DQ493256	A/chicken/Guizhou/7/2008(H5N1)	HM172323
A/chicken/Nigeria/1071‐4/2007(H5N1)	EU148377	A/chicken/Hebei/A‐8/2009(H5N1)	HM172324
A/chicken/Yamaguchi/7/2004(H5N1)	AB166861	A/chicken/Jiangsu/18/2008(H5N1)	HM172352
A/China/GD01/2006(H5N1)	DQ835312	A/duck/Eastern China/909/2009(H5N1)	GU727676
A/crested eagle/Belgium/01/2004(H5N1)	DQ454103	A/duck/Hunan/S4220/2011(H5N1)	CY146699
A/duck/Indonesia/MS/2004(H5N1)	AY651600	A/barn swallow/Hong Kong/1161/2010(H5N1)	KF735643
A/Dk/Viet Nam/11/2004(H5N1)	AY651621	A/duck/Potsdam/2216‐4/1984(H5N6)	CY005774
A/domestic mallard duck/Hunan/67/2005(H5N1)	EU430501	A/mallard/Wisconsin/34/1975(H5N6)	GU052003
A/domestic mallard duck/Hunan/79/2005(H5N1)	EU430508	A/environment/Jiangxi/10170/2014(H5N6)	KP286460
A/duck/Hubei/wq/2003(H5N1)	DQ997176	A/cat/Sichuan/SC18/2014(H5N6)	KM873637
A/duck/Hunan/856/2006(H5N1)	EF124708	A/swan goose/Jilin/JL01/2014(H5N6)	KM873645
A/Goose/Guangdong/1/1996(H5N1)	NC_007359	A/duck/Jiangxi/NCDZT1126/2014(H5N6)	KP090446
A/Grebe/Tyva/Tyv06‐1/2006(H5N1)	DQ978999	A/duck/Sichuan/NCXJ16/2014(H5N6)	KM251516
A/Hong Kong/156/1997(H5N1)	AF084267	A/chicken/Dongguan/3363/2013(H5N6)	KP286108
A/Hong Kong/483/1997(H5N1)	GU052102	A/environment/Jiangxi/10171/2014(H5N6)	KP285052
A/Hong Kong/97/98(H5N1)	AF257202	A/chicken/Shenzhen/552/2013(H5N6)	KP284964
A/House crow/Hong Kong/2648/2006(H5N1)	EF124649	A/swine/Guangdong/2/2014(H5N6)	KT313415
A/Indonesia/292H/2006(H5N1)	EU146718	A/muscovy A/duck/Vietnam/HU1‐1144/2014(H5N6)	LC069898
A/Indonesia/CDC1031/2007(H5N1)	CY019351	A/chicken/Zhejiang/727155/2014(H5N6)	KU042737
A/Thailand/676/2005(H5N1)	DQ360839	A/turtledove/Wuhan/HKBJ07/2015(H5N6)	KU143484
A/Tiger/Thailand/VSMU‐1‐SPB/2004(H5N1)	EF178524	A/chicken/Wuhan/WHYJ01/2015(H5N6)	KU143491
A/duck/Hunan/689/2006(H5N1)	FJ784793	A/duck/Taizhou/TZYG12/2015(H5N6)	KU143495
A/duck/Hunan/3315/2006(H5N1)	FJ784807	A/Guangzhou/39715/2014(H5N6)	KP765787
A/chicken/Henan/1362/2006(H5N1)	FJ784795	A/Yunnan/0127/2015(H5N6)	KT245148
A/duck/Henan/1650/2006(H5N1)	FJ784797	A/Sichuan/26221/2014(H5N6)	EPI533587*
A/duck/Hunan/1930/2007(H5N1)	FJ784800	A/quail/Jiangxi/B9/2015(H5N6)	EPI1574328*
A/duck/Hunan/3340/2006(H5N1)	FJ784808	A/Streptopelia decaocto/Jiangxi/E1/2015(H5N6)	EPI1574312*
A/chicken/Hong Kong/947/2006(H5N1)	EF124658	A/Streptopelia decaocto/Jiangxi/G6/2016(H5N6)	EPI1489547*
A/Goose/Guiyang/1794/2006(H5N1)	EF124774	A/Mallard/Jiangxi/JXH9/2014(H5N6)	EPI1574344*
A/Guinea fowl/Shantou/1341/2006(H5N1)	EF124762	A/Mallard/Jiangxi/JXH22/2014(H5N6)	EPI1574336*
A/Migratory duck/Jiangxi/2136/2005(H5N1)	DQ321248	A/Streptopelia decaocto/Jiangxi/J8/2015(H5N6)	EPI1574320*
A/Munia/Hong Kong/2454/2006(H5N1)	EF124646	A/Luscinia cyane/Jiangxi/U2/2016(H5N6)	EPI1574304*
A/great crested‐grebe/Qinghai/1/2009(H5N1)	CY063317	A/duck/Jiangxi/95/2014(H5N6)	EPI530053*
A/bar‐headed goose/Mongolia/X53/2009(H5N1)	KR732516	A/duck/Jiangxi/E7/2014(H5N6)	Unpublished
A/bean goose/Tyva/10/2009(H5N1)	GQ386153	A/chicken/Jiangxi/E84/2014(H5N6)	Unpublished
A/environment/Hunan/6‐69/2008(H5N1)	GU182181	A/duck/Jiangxi/F31/2014(H5N6)	Unpublished
A/grey heron/Hong Kong/1046/2008(H5N1)	CY036244	A/mallard/Jiangxi/G98/2014(H3N8)	MN473212
A/whooper swan/Hokkaido/1/2008(H5N1)	AB436549	A/swan goose/Jiangxi/H24/2014(H3N6)	MT375534
A/common magpie/Hong Kong/5052/2007(H5N1)	CY036172	A/swan goose/Jiangxi/H23/2014(H3N6)	MT375332

* Data source: GISAID.

**TABLE A6:** Details of sequences of NP gene analysed in this study

Viruses name	Accession	Viruses name	Accession
A/VietNam/1203/2004(H5N1)	HM006760	A/house crow/Hong Kong/1203/2007(H5N1)	CY036102
A/Bar‐headed Goose/Qinghai/59/05(H5N1)	DQ095672	A/little egret/Hong Kong/8550/2007(H5N1)	CY036174
A/Brown‐headed Gull/Qinghai/3/05(H5N1)	DQ095676	A/peregrine falcon/Hong Kong/1143/2007(H5N1)	CY036094
A/chicken/Egypt/2253‐1/2006(H5N1)	CY020648	A/peregrine falcon/Hong Kong/2142/2008(H5N1)	CY036270
A/chicken/Guiyang/3570/2005(H5N1)	EF124459	A/peregrine falcon/Hong Kong/5211/2006(H5N1)	CY036046
A/chicken/HongKong/282/2006(H5N1)	EF124351	A/chicken/Hebei/A‐8/2009(H5N1)	HM172217
A/chicken/Korea/IS/2006(H5N1)	EU233678	A/chicken/Hunan/1/2009(H5N1)	HM172255
A/chicken/Magetan/BBVW/2005(H5N1)	DQ493080	A/chicken/Hunan/8/2008(H5N1)	GU182159
A/chicken/Malaysia/5858/2004(H5N1)	DQ321132	A/great crested‐grebe/Qinghai/1/2009(H5N1)	CY063319
A/chicken/Nigeria/1071‐4/2007(H5N1)	EU148375	A/tree sparrow/Jiangsu/1/2008(H5N1)	GQ202210
A/chicken/Yamaguchi/7/2004(H5N1)	AB166863	A/duck/Jiangxi/95/2014(H5N6)	EPI530055*
A/China/GD01/2006(H5N1)	DQ835314	A/duck/Potsdam/2216‐4/1984(H5N1)	CY005772
A/crested eagle/Belgium/01/2004(H5N1)	DQ454104	A/duck/Hunan/S4220/2011(H5N1)	CY146701
A/duck/Indonesia/MS/2004	AY651486	A/duck/Zhejiang/2248/2011(H5N1)	JN646726
A/Dk/Viet Nam/11/2004(H5N1)	AY651509	A/mallard/Wisconsin/34/1975(H5N6)	GU052002
A/domestic mallard duck/Hunan/79/2005(H5N1)	EU430504	A/cat/Sichuan/SC18/2014(H5N6)	KM873639
A/duck/Guangdong/01/2001(H5N1)	AY585424	A/swan goose/Jilin/JL01/2014(H5N6)	KM873647
A/duck/Guangxi/793/2005(H5N1)	DQ321096	A/duck/Jiangxi/NCDZT1123/2014(H5N6)	KP090440
A/duck/Guangxi/2143/2006(H5N1)	EF124490	A/duck/Jiangxi/NCDZT1126/2014(H5N6)	KP090448
A/duck/Hubei/wq/2003(H5N1)	DQ997174	A/duck/Sichuan/NCXJ16/2014(H5N6)	KM251496
A/duck/Hunan/856/2006(H5N1)	EF124406	A/chicken/Shenzhen/552/2013(H5N6)	KP284966
A/egret/Hong Kong/757.2/2003(H5N1)	AY676038	A/environment/Jiangxi/10171/2014(H5N6)	KP285054
A/grebe/Tyva/Tyv06‐1/2006(H5N1)	DQ916293	A/environment/Jiangxi/10170/2014(H5N6)	KP286462
A/HongKong/156/1997(H5N1)	GU052130	A/duck/Jiangxi/13469/2014(H5N6)	KP286422
A/Hong Kong/483/1997(H5N1)	GU052100	A/duck/Jiangxi/13475/2014(H5N6)	KP286430
A/Hong Kong/97/98(H5N1)	AF255753	A/duck/Jiangxi/13484/2014(H5N6)	KP286438
A/house crow/Hong Kong/2648/2006(H5N1)	EF124347	A/chicken/Dongguan/3363/2013(H5N6)	KP286110
A/Indonesia/292H/2006(H5N1)	EU146716	A/swine/Guangdong/2/2014(H5N6)	KT313413
A/Indonesia/CDC1031/2007(H5N1)	CY019353	A/chicken/Wuhan/WHYJ01/2015(H5N6)	KU143401
A/Thailand/676/2005(H5N1)	DQ360838	A/duck/Taizhou/TZYG12/2015(H5N6)	KU143406
A/Tiger/Thailand/VSMU‐1‐SPB/2004(H5N1)	EF178525	A/turtledove/Wuhan/HKBJ07/2015(H5N6)	KU143408
A/duck/Hunan/689/2006(H5N1)	FJ784777	A/Sichuan/26221/2014(H5N6)	EPI533588
A/chicken/Henan/1362/2006(H5N1)	FJ784779	A/Guangzhou/39715/2014(H5N6)	KP765789
A/duck/Hunan/1930/2007(H5N1)	FJ784784	A/Yunnan/0127/2015(H5N6)	KT245146
A/duck/Hunan/3340/2006(H5N1)	FJ784792	A/quail/Jiangxi/B9/2015(H5N6)	EPI1574330*
A/chicken/HongKong/947/2006(H5N1)	EF124356	A/Streptopelia decaocto/Jiangxi/E1/2015(H5N6)	EPI1574314*
A/goose/Shantou/239/2006(H5N1)	EF124462	A/Streptopelia decaocto/Jiangxi/G6/2016(H5N6)	EPI1489549*
A/guinea fowl/Shantou/1341/2006(H5N1)	EF124460	A/Mallard/Jiangxi/JXH9/2014(H5N6)	EPI1574346*
A/migratory duck/Jiangxi/2136/2005(H5N1)	DQ321116	A/Mallard/Jiangxi/JXH22/2014(H5N6)	EPI1574338*
A/munia/Hong Kong/2454/2006(H5N1)	EF124344	A/Streptopelia decaocto/Jiangxi/J8/2015(H5N6)	EPI1574322*
A/chicken/Rostock/45/1934(H7N1)	CY015049	A/Luscinia cyane/Jiangxi/U2/2016(H5N6)	EPI1574306*
A/bar‐headed goose/Mongolia/X53/2009(H5N1)	KR732429	A/duck/Jiangxi/95/2014(H5N6)	EPI530055*
A/bean goose/Tyva/10/2009(H5N1)	GQ386152	A/duck/Jiangxi/E7/2014(H5N6)	Unpublished
A/environment/Hunan/6‐69/2008(H5N1)	GU182183	A/chicken/Jiangxi/E84/2014(H5N6)	Unpublished
A/greyheron/HongKong/1046/2008(H5N1)	CY036246	A/duck/Jiangxi/F31/2014(H5N6)	Unpublished
A/whooper swan/Hokkaido/1/2008(H5N1)	AB436551	A/mallard/Jiangxi/G98/2014(H3N8)	MN473214
A/black‐crowned night heron/Hong Kong/659/2008(H5N1)	CY036230	A/swan goose/Jiangxi/H24/2014(H3N6)	MT375536
A/common magpie/Hong_Kong/5052/2007(H5N1)	CY036174	A/swan goose/Jiangxi/H23/2014(H3N6)	MT375334
A/crested goshawk/Hong Kong/458/2007(H5N1)	CY036062		

* Data source: GISAID.

**TABLE A7:** Details of sequences of M gene analysed in this study

Viruses name	Accession	Viruses name	Accession
A/domestic mallard duck/Hunan/79/2005(H5N1)	EU430507	A/chicken/Shandong/zc11/2009(H9N2)	KC821184
A/Goose/Guangdong/1/1996(H5N1)	NC_007363	A/muscovy duck/Viet Nam/TMU012/2008(H5N1)	CY103815
A/chicken/Rostock/45/1934(H7N1)	CY077423	A/chicken/Laos/Xaythiani 26/2006(H5N1)	HM006754
A/mallard/Netherlands/12/2000(H7N3)	KF695242	A/chicken/Viet Nam/TMU008/2008(H5N1)	CY103809
A/chicken/Hong Kong/947/2006(H5N1)	EF124054	A/duck/Hunan/3315/2006(H5N1)	FJ784887
A/chicken/Guizhou/7/2008(H5N1)	HM172157	A/chicken/Hubei/2856/2007(H5N1)	FJ784883
A/chicken/Hunan/8/2008(H5N1)	GU182162	A/duck/Henan/1650/2006(H5N1)	FJ784877
A/great crested‐grebe/Qinghai/1/2009(H5N1)	CY063321	A/duck/Cambodia/191W5M4/2013(H5N1)	KF001445
A/grey heron/Hong Kong/1046/2008(H5N1)	CY036248	A/duck/Vietnam/LBM633/2014(H5N1)	AB979474
A/little egret/Hong Kong/8550/2007(H5N1)	CY036200	A/egret/Hong Kong/757.2/2003(H5N1)	AY676046
A/duck/Potsdam/2216‐4/1984(H5N6)	CY005770	A/parrot/Guangdong/C99/2005(H5N1)	JX013491
A/mallard/Wisconsin/34/1975(H5N6)	GU052001	A/water/Hunan/3/2009(H5N1)	CY098848
A/duck/Jiangxi/95/2014(H5N6)	EPI530057	A/duck/Jiangxi/10160/2014(H5N6)	KP285040
A/duck/Hunan/S4220/2011(H5N1)	CY146703	A/environment/Jiangxi/10164/2014(H5N6)	KP285048
A/Shenzhen/1/2011(H5N1)	KC436112	A/environment/Jiangxi/10171/2014(H5N6)	KP285056
A/Sichuan/26221/2014(H5N6)	EPI533589*	A/Yunnan/0127/2015(H5N6)	KT245144
A/duck/Vietnam/LBM360c1‐4‐1/2013(H5N6)	LC010699	A/swine/Guangdong/1/2014(H5N6)	KT313403
A/duck/Laos/XBY004/2014(H5N6)	KM496980	A/swine/Guangdong/2/2014(H5N6)	KT313411
A/chicken/Laos/LPQ001/2014(H5N6)	KM496968	A/quail/Jiangxi/B9/2015(H5N6)	EPI1574332*
A/environment/Zhenjiang/C13/2013(H5N6)	KJ938661	A/Streptopelia decaocto/Jiangxi/E1/2015(H5N6)	EPI1574316*
A/duck/Guangdong/GD01/2014(H5N6)	KJ754148	A/Streptopelia decaocto/Jiangxi/G6/2016(H5N6)	EPI1489551*
A/environment/Shenzhen/25‐24/2013(H5N6)	EPI530066*	A/Mallard/Jiangxi/JXH9/2014(H5N6)	EPI1574348*
A/muscovy duck/Vietnam/LBM227/2012(H5N1)	AB786679	A/Mallard/Jiangxi/JXH22/2014(H5N6)	EPI1574340*
A/Chinese pond heron/Jiangxi/B4/2013	Unpublished	A/Streptopelia decaocto/Jiangxi/J8/2015(H5N6)	EPI1574324*
A/duck/Jiangxi/1/2009	Unpublished	A/Luscinia cyane/Jiangxi/U2/2016(H5N6)	EPI1574308*
A/duck/Jiangxi/28/2009	Unpublished	A/duck/Jiangxi/95/2014(H5N6)	EPI530057*
A/duck/Jiangxi/145/2010	Unpublished	A/duck/Jiangxi/E7/2014(H5N6)	Unpublished
A/duck/Jiangxi/9078/2010	Unpublished	A/chicken/Jiangxi/E84/2014(H5N6)	Unpublished
A/chicken/Jiangxi/T1/2013	Unpublished	A/duck/Jiangxi/F31/2014(H5N6)	Unpublished
A/chicken/Shandong/JL/2008(H9N2)	KC821179	A/swan goose/Jiangxi/H24/2014(H3N6)	MT375538
A/environment/Hunan/5‐38/2007(H9N2)	GU474609	A/swan goose/Jiangxi/H23/2014(H3N6)	MT375336
A/chicken/Shandong/B4/2007(H9N2)	EU532033	A/mallard/Jiangxi/G98/2014(H3N8)	MN473216
A/chicken/Guangxi/55/2005(H9N2)	EU086248		

* Data source: GISAID.

**TABLE A8:** Details of sequences of NS gene analysed in this study

Viruses name	Accession	Viruses name	Accession
A/VietNam/1203/2004(H5N1)	HM006763	A/house crow/Hong Kong/1203/2007(H5N1)	CY036201
A/Bar‐headed Goose/Qinghai/59/05(H5N1)	DQ095692	A/little egret/Hong Kong/8550/2007(H5N1)	CY036105
A/Brown‐headed Gull/Qinghai/3/05(H5N1)	DQ095696	A/peregrine falcon/Hong Kong/1143/2007(H5N1)	CY036097
A/chicken/Egypt/2253‐1/2006(H5N1)	CY020649	A/peregrine falcon/Hong Kong/2142/2008(H5N1)	CY036273
A/chicken/Guiyang/3570/2005(H5N1)	EF124610	A/peregrine falcon/Hong Kong/5211/2006(H5N1)	CY036049
A/chicken/Korea/IS/2006(H5N1)	EU233679	A/chicken/Guizhou/7/2008(H5N1)	HM172279
A/chicken/Magetan/BBVW/2005(H5N1)	DQ493168	A/chicken/Jiangsu/18/2008(H5N1)	HM172275
A/chicken/Malaysia/5858/2004(H5N1)	DQ321197	A/duck/Potsdam/2216‐4/1984(H5N6)	CY005773
A/chicken/Nigeria/1071‐4/2007(H5N1)	EU148376	A/duck/Hunan/S4220/2011(H5N1)	CY146704
A/chicken/Yamaguchi/7/2004(H5N1)	AB166866	A/barn swallow/Hong Kong/1161/2010(H5N1)	KF735646
A/China/GD01/2006(H5N1)	DQ835317	A/mallard/Wisconsin/34/1975(H5N6)	U85379
A/chicken/ST/4231/2003(H5N1)	AY651585	A/cat/Sichuan/SC18/2014(H5N6)	KM873642
A/crested eagle/Belgium/01/2004(H5N1)	DQ454105	A/swan goose/Jilin/JL01/2014(H5N6)	KM873650
A/duck/Indonesia/MS/2004(H5N1)	AY651540	A/duck/Jiangxi/NCDZT1126/2014(H5N6)	KP090451
A/Dk/Viet Nam/11/2004(H5N1)	AY651563	A/duck/Sichuan/NCXJ16/2014(H5N6)	KM251506
A/domestic mallard duck/Hunan/67/2005(H5N1)	EU430503	A/environment/Jiangxi/10171/2014(H5N6)	KP285057
A/domestic mallard duck/Hunan/79/2005(H5N1)	EU430506	A/duck/Jiangxi/13484/2014(H5N6)	KP286441
A/duck/Guangxi/793/2005(H5N1)	DQ321162	A/chicken/Dongguan/3363/2013(H5N6)	KP286113
A/duck/Guangxi/2143/2006(H5N1)	EF124641	A/swine/Guangdong/2/2014(H5N6)	KT313414
A/duck/Hubei/wq/2003(H5N1)	DQ997175	A/chicken/Zhejiang/727155/2014(H5N6)	KU042882
A/duck/Hunan/182/2005(H5N1)	DQ321172	A/turtledove/Wuhan/HKBJ07/2015(H5N6)	KU143440
A/duck/Hunan/856/2006(H5N1)	EF124557	A/duck/Taizhou/TZYG12/2015(H5N6)	KU143452
A/Goose/Guangdong/1/1996(H5N1)	NC_007364	A/Guangzhou/39715/2014(H5N6)	KP765792
A/grebe/Tyva/Tyv06‐1/2006(H5N1)	DQ914806	A/Yunnan/0127/2015(H5N6)	KT245147
A/grey heron/Hong Kong/728/2004(H5N1)	DQ321186	A/duck/Guangdong/GD01/2014(H5N6)	KJ754149
A/Hong Kong/156/1997(H5N1)	AF036360	A/duck/Vietnam/LBM759/2014(H5N6)	LC028343
A/Hong Kong/483/1997(H5N1)	GU052101	A/goose/Shantou/1791/2014(H5N6)	KP285329
A/Hong Kong/97/98(H5N1)	AF256188	A/duck/Wenzhou/YHQL22/2014(H5N6)	KU143450
A/Indonesia/292H/2006(H5N1)	EU146717	A/Sichuan/26221/2014(H5N6)	EPI533590*
A/Indonesia/CDC1031/2007(H5N1)	CY019356	A/duck/Zhejiang/W24/2013(H5N8)	KJ476677
A/Quail/Shantou/911/05(H5N1)	DQ095707	A/goose/Shandong/WFSG1/2014(H5N8)	KT221078
A/Thailand/676/2005(H5N1)	DQ360836	A/duck/Zhejiang/6D18/2013(H5N8)	KJ476678
A/Tiger/Thailand/VSMU‐1‐SPB/2004(H5N1)	EF178527	A/duck/Japan/11UO0012/2011(H5N2)	KR265588
A/mallard/Netherlands/12/00(H7N3)	CY005848	A/duck/Zhejiang/925169/2014(H5N8)	KU042884
A/chicken/Henan/1362/2006(H5N1)	FJ784859	A/quail/Jiangxi/B9/2015(H5N6)	EPI1574333*
A/duck/Hunan/1930/2007(H5N1)	FJ784864	A/Streptopelia decaocto/Jiangxi/E1/2015(H5N6)	EPI1574317*
A/duck/Hunan/3340/2006(H5N1)	FJ784872	A/Streptopelia decaocto/Jiangxi/G6/2016(H5N6)	EPI1489552*
A/domestic green‐winged teal/Hunan/3450/2006(H5N1)	KC690160	A/Mallard/Jiangxi/JXH9/2014(H5N6)	EPI1574349*
A/duck/Hunan/689/2006(H5N1)	FJ784857	A/Mallard/Jiangxi/JXH22/2014(H5N6)	EPI1574340*
A/chicken/Hong Kong/947/2006(H5N1)	EF124507	A/Streptopelia decaocto/Jiangxi/J8/2015(H5N6)	EPI1574325*
A/goose/Guangxi/345/2005(H5N1)	DQ321159	A/Luscinia cyane/Jiangxi/U2/2016(H5N6)	EPI1574309*
A/goose/Guiyang/1794/2006(H5N1)	EF124623	A/duck/Jiangxi/95/2014(H5N6)	EPI530058*
A/goose/Shantou/239/2006(H5N1)	EF124613	A/duck/Jiangxi/E7/2014(H5N6)	Unpublished
A/guinea fowl/Shantou/1341/2006(H5N1)	EF124611	A/chicken/Jiangxi/E84/2014(H5N6)	Unpublished
A/migratory duck/Jiangxi/2136/2005(H5N1)	DQ321182	A/duck/Jiangxi/F31/2014(H5N6)	Unpublished
A/munia/Hong Kong/2454/2006(H5N1)	EF124495	A/swan goose/Jiangxi/H24/2014(H3N6)	MT375539
A/wild duck/Hunan/211/2005(H5N1)	EU329182	A/swan goose/Jiangxi/H23/2014(H3N6)	MT375337
A/great crested‐grebe/Qinghai/1/2009(H5N1)	CY063322	A/mallard/Jiangxi/G98/2014(H3N8)	MN473217
A/bar‐headed goose/Mongolia/X53/2009(H5N1)	KR732555	A/common teal/Nanji/NJ‐233/2013(H6N1)	KJ907667
A/bean goose/Tyva/10/2009(H5N1)	GQ386157	A/waterfowl/Korea/S353/2016(H11N9)	KX703023
A/chicken/Hunan/8/2008(H5N1)	GU182161	A/duck/Hubei/HF5/2017(H7N8)	MH458926
A/environment/Hunan/6‐69/2008(H5N1)	GU182186	A/Pigeon/Longquan/LQ67/2016(H2N8)	KY415936
A/greyheron/HongKong/1046/2008(H5N1)	CY036249	A/duck/Zhejiang/77139/2014(H4N2)	KT589301
A/whooper swan/Hokkaido/1/2008(H5N1)	AB436554	A/white‐fronted goose/Korea/F56‐3/2017(H6N2)	MH130150
A/common magpie/Hong Kong/5052/2007(H5N1)	CY036177	A/wild waterfowl/Korea/F56‐1/2017(H6N2)	MH130142
A/crested goshawk/Hong Kong/458/2007(H5N1)	CY036065	A/duck/Fujian/JF47/2014(H1N1)	KP658071

* Data source: GISAID.

**TABLE A9:** Comparison of different nucleotide substitution model

HA		NA		PB2		PB1	
Model	BIC	Model	BIC	Model	BIC	Model	BIC
GTR + G	22,739	TN93 + G	24,021	GTR + G + I	34,538	TN93 + G	32,612
GTR + G + I	22,747	TN93 + G + I	24,025	GTR + G	34,546	K2 + G + I	32,612
HKY + G	22,759	HKY + G	24,033	K2 + G + I	34,548	GTR + G	32,620
K2 + G	22,763	K2 + G + I	24,034	T93 + G	34,649	HKY + G	32,624
K2 + G + I	22,768	HKY + G + I	24,037	K2 + G	34,710	HKY + G + I	32,624

PA		NP		M		NS	
Model	BIC	Model	BIC	Model	BIC	Model	BIC
K2 + G + I	28,477	K2 + G + I	19,604	K2 + G	9,630	K2 + G + I	14,602
HKY + G	28,483	HKY + G	19,611	T92 + G	9,634	HKY + G	14,605
TN93 + G	28,486	TN93 + G + I	19,616	K2 + G + I	9,636	HKY + G + I	14,613
GTR + G	28,489	T92 + G	19,622	HKY + G	9,640	T92 + G	14,613
K2 + G	28,495	K2 + I	19,623	GTR + G	9,641	GTR + G	14,616

Note

The performance of the General Time Reversible (GTR), Hasegawa‐Kishino‐Yano (HKY), Tamura‐Nei (TN93), Tamura 3‐parameter (T92), Kimura 2‐parameter (K2) and Jukes‐Cantor (JC) model were compared. Evolutionary rates among sites were modelled by discrete Gamma distribution (+G), assuming a certain fraction of invariant sites (+I). The top‐5 models and corresponding BIC scores for each gene were presented. For each gene, the performance of the top‐5 optimal models varies minimally. The widely‐used Kimura two‐parameter model with the non‐uniform distribution of evolutionary rates among sites (K2 + G + I) was applied to our analysis.

## References

[mbo31060-bib-0001] Bean, W. J. , Schell, M. , Katz, J. , Kawaoka, Y. , Naeve, C. , Gorman, O. , & Webster, R. G. (1992). Evolution of the H3 influenza virus hemagglutinin from human and nonhuman hosts. Journal of Virology, 66, 1129–1138. 10.1128/JVI.66.2.1129-1138.1992 1731092 PMC240817

[mbo31060-bib-0002] Bi, Y. , Chen, Q. , Wang, Q. , Chen, J. , Jin, T. , Wong, G. , … Gao, G. F. (2016). Genesis, evolution and prevalence of H5N6 avian influenza viruses in China. Cell Host & Microbe, 20, 810–821. 10.1016/j.chom.2016.10.022 27916476

[mbo31060-bib-0003] Bi, Y. , Lu, L. , Li, J. , Yin, Y. , Zhang, Y. , Gao, H. , … Liu, W. (2011). Novel genetic reassortants in H9N2 influenza A viruses and their diverse pathogenicity to mice. Virology Journal, 8, 505 10.1186/1743-422X-8-505 22050764 PMC3236014

[mbo31060-bib-0004] Cui, H. , Shi, Y. , Ruan, T. , Li, X. , Teng, Q. , Chen, H. , … Li, Z. (2016). Pathogenetic analysis and pathogenicity of H3 subtype avian influenza viruses isolated from live poultry markets in China. Scientific Reports, 6, 27360. 27270298 10.1038/srep27360 PMC4895239

[mbo31060-bib-0005] Deng, G. , Tan, D. , Shi, J. , Cui, P. , Jiang, Y. , Liu, L. , … Chen, H. (2013). Complex reassortment of multiple subtypes of avian influenza viruses in domestic ducks at the Dongting Lake region of China. Journal of Virology, 87, 9452–9462. 10.1128/JVI.00776-13 23804642 PMC3754128

[mbo31060-bib-0006] Hoffmann, E. , Stech, J. , Guan, Y. , Webster, R. G. , & Perez, D. R. (2001). Universal primer set for the full‐length amplification of all influenza A viruses. Archives of Virology, 146, 2275–2289. 10.1007/s007050170002 11811679

[mbo31060-bib-0007] Kimura, M. (1980). A simple method for estimating evolutionary rate of base substitutions through comparative studies of nucleotide sequences. Journal of Molecular Evolution, 16, 111–120. 7463489 10.1007/BF01731581

[mbo31060-bib-0008] Lee, M. S. , Chang, P. C. , Shien, J. H. , Cheng, M. C. , & Shieh, H. K. (2001). Identification and subtyping of avian influenza viruses by reverse transcription‐PCR. Journal of Virological Methods, 97, 13–22. 10.1016/S0166-0934(01)00301-9 11483213

[mbo31060-bib-0009] Li, X. , Yang, J. , Liu, B. , Jia, Y. , Guo, J. , Gao, X. , … Zhu, Q. (2016). Co‐circulation of H5N6, H3N2, H3N8, and emergence of novel reassortant H3N6 in a local community in Hunan Province in China. Scientific Reports, 6, 25549 10.1038/srep25549 27151540 PMC4858758

[mbo31060-bib-0010] Liu, D. , Shi, W. , & Gao, G. F. (2014). Poultry carrying H9N2 act as incubators for novel human avian influenza viruses. Lancet, 383, 869. 24581684 10.1016/S0140-6736(14)60386-X

[mbo31060-bib-0011] Liu, M. , Li, X. , Yuan, H. , Zhou, J. , Wu, J. , Bo, H. , … Shu, Y. (2015). Genetic diversity of avian influenza A(H10N8) virus in live poultry markets and its association with human infections in China. Scientific Reports, 15(5), 7632 10.1038/srep07632 PMC5379002 25591167

[mbo31060-bib-0012] Liu, T. , Xie, Z. , Luo, S. , Xie, L. , Deng, X. , Xie, Z. , … Wang, S. (2015). Characterization of the whole‐genome sequence of an H3N6 avian influenza virus, isolated from a domestic duck in Guangxi, southern China. Genome Announcements, 3, e01190–e1215. 10.1128/genomeA.01190-15 26472834 PMC4611686

[mbo31060-bib-0013] Luo, S. , Xie, Z. , Xie, Z. , Xie, L. , Huang, L. , Huang, J. , … Liu, J. (2017). Surveillance of live poultry markets for low pathogenic avian influenza viruses in Guangxi Province, southern China, from 2012–2015. Scientific Reports, 7, 17577. 29242521 10.1038/s41598-017-17740-0 PMC5730573

[mbo31060-bib-0014] Pu, J. , Wang, S. , Yin, Y. , Zhang, G. , Carter, R. A. , Wang, J. , … Webster, R. G. (2015). Evolution of the H9N2 influenza genotype that facilitated the genesis of the novel H7N9 virus. Proceedings of the National Academy of Sciences of the United States of America, 112(2), 548–553. 10.1073/pnas.1422456112 25548189 PMC4299237

[mbo31060-bib-0015] Tamura, K. , Stecher, G. , Peterson, D. , Filipski, A. , & Kumar, S. (2013). MEGA6: Molecular evolutionary genetics analysis version 6.0. Molecular Biology and Evolution, 30, 2725–2729. 10.1093/molbev/mst197 24132122 PMC3840312

[mbo31060-bib-0016] Tsukamoto, K. , Ashizawa, T. , Nakanishi, K. , Kaji, N. , Suzuki, K. , Shishido, M. , … Mase, M. (2009). Use of reverse transcriptase PCR to subtype N1 to N9 neuraminidase genes of avian influenza viruses. Journal of Clinical Microbiology, 47, 2301–2303. 19403772 10.1128/JCM.02366-08 PMC2708491

[mbo31060-bib-0018] Zhang, T. , Li, R. , Zhu, G. , Chang, J. , & Xu, B. (2019). First detection of a novel reassortant avian influenza A(H5N6) clade 2.3.2.1c virus, isolated from a wild bird in China. Microbiology Resource Announcements, 8, e00797–e819. 10.1128/MRA.00797-19 31488532 PMC6728642

